# Unique pharmacological properties of etrasimod among S1P receptor modulators

**DOI:** 10.1002/2211-5463.13907

**Published:** 2024-11-20

**Authors:** Ibragim Gaidarov, H. Kiyomi Komori, Dariusz T. Stepniak, Karin Bruinsma, Huong Dang, Xiaohua Chen, Todd Anthony, Joel Gatlin, Lisa Karimi‐Naser, Anh‐Tuan Ton, Tim Indersmitten, Paul E. Miller, Andre Ghetti, Najah Abi‐Gerges, David Unett, Hussien Al‐Shamma, Christopher J. Rabbat, Catherine Crosby, John W. Adams

**Affiliations:** ^1^ Beacon Discovery San Diego CA USA; ^2^ Arena Pharmaceuticals San Diego CA USA; ^3^ AnaBios Corporation San Diego CA USA

**Keywords:** etrasimod, pharmacodynamics, pharmacokinetics, sphingosine 1‐phosphate receptor modulator

## Abstract

Etrasimod (ADP334) is an oral, once‐daily, selective sphingosine 1‐phosphate (S1P)_1,4,5_ receptor modulator for the treatment of moderately to severely active ulcerative colitis and in development for the treatment of immune‐mediated inflammatory diseases. Interaction between S1P and its five receptor subtypes (S1P_1_–S1P_5_) plays a role in several physiologic systems, including the cardiovascular and immune systems. Since differences in S1PR binding and downstream intracellular signaling could contribute to distinct profiles of drug efficacy and safety, we directly compared the S1P_1–5_ selectivity profile of etrasimod to three marketed S1PR modulators: fingolimod, ozanimod, and siponimod. Using both heterologous expression systems and human umbilical vein endothelial cells that spontaneously express S1P_1_, we profiled key S1P_1_ downstream signaling pathways and found that etrasimod had similar potency to the other tested S1PR modulators in promoting β‐arrestin recruitment and S1P_1_ internalization. However, etrasimod was notably less potent than other S1PR modulators in assays measuring S1P_1_‐mediated G protein activation (GTPγS binding and cAMP inhibition). Relatively lower potency of etrasimod in inducing G protein signaling corresponded to significantly diminished activation of human cardiac G protein‐coupled inwardly rectifying potassium channels when compared to ozanimod. Together with pharmacokinetic properties, this pharmacologic profile of etrasimod may contribute to the positive benefit risk profile of etrasimod observed during the phase III ELEVATE UC 52 and ELEVATE UC 12 trials in patients with moderately to severely active ulcerative colitis.

## Graphical abstract

Several S1P receptor modulators are being studied for treating immune‐mediated inflammatory diseases, each showing distinct selectivity and signaling activities. Downstream of S1P_1_, etrasimod showed similar potency to other S1P receptor modulators in promoting β‐arrestin recruitment and S1P_1_ internalization but was less potent in G protein activation, which may contribute to its favorable benefit–risk profile in treating conditions such as ulcerative colitis.

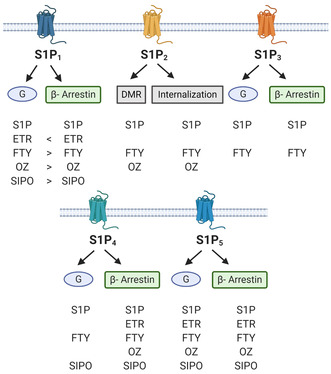

**Abbreviations**
BSA: bovine serum albumin
DMSO: dimethyl sulfoxide
EC_50_
: half maximal effective concentration
ECGM: endothelial cell growth medium
FBS: fetal bovine serum
fingolimod(P): fingolimod phosphate
GIRK: G protein‐coupled inwardly rectifying potassium
GPCR: G protein‐coupled receptor
HA: hemagglutinin
HUVEC: human umbilical vein endothelial cells
NK: natural killer
ozanimod(k): ozanimod ketone
PBS: phosphate‐buffered saline
RA: right atria
S1P: sphingosine 1‐phosphate
S1PR: sphingosine 1‐phosphate receptor

Sphingosine 1‐phosphate (S1P) is a natural phospholipid that exerts its biological functions by signaling through five‐cell surface‐expressed G protein‐coupled receptors (GPCRs), sphingosine 1‐phosphate receptors 1 through 5 (S1P_1–5_; Fig. 1) [1, 2, 3, 4]. Virtually, all cell types express one or a combination of these receptors, which can activate either distinct or overlapping signaling pathways. Among S1P receptors, S1P_1_, S1P_2_, and S1P_3_ are ubiquitously expressed, while expression of S1P_4_ is largely restricted to immune cells and S1P_5_ is selectively expressed on natural killer (NK) cells, T cells, oligodendrocytes, and keratinocytes [5, 6, 7, 8]. The cellular outcomes of S1PR activation depend on particular cell types and the signaling strength [9].

**Fig. 1:**
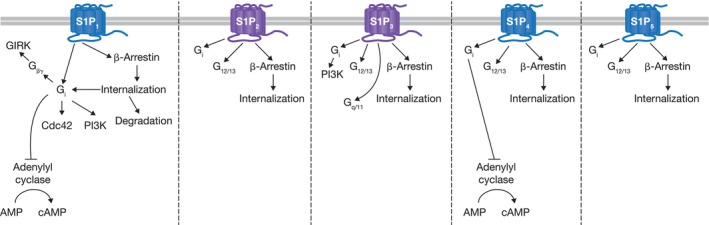
S1P receptor downstream signaling. Etrasimod selectively activates S1P_1_, S1P_4_, and S1P_5_ (colored blue), with no detectable activity on S1P_2_ or S1P_3_ (colored purple). AMP, adenosine monophosphate; cAMP, cyclic adenosine monophosphate; Cdc42, Cell division control protein 42 homolog; GIRK, G protein‐coupled inwardly rectifying potassium; PI3K, phosphoinositide 3‐kinase; S1P, sphingosine 1‐phosphate.

A well‐characterized function of the S1P/S1PR axis is regulation of lymphocyte migration. Stimulation of S1P_1_ activates both G_i_ and β‐arrestin‐related pathways, which promote the egress of lymphocytes from lymph nodes or drive the internalization, degradation of S1P, and recycling of the S1P_1_ receptor back to the cell surface, respectively [9, 10]. Disruption of S1P_1_ signaling occurs via T‐cell activation through the downregulation of S1P_1_, which subsequently retains newly activated lymphocytes in the lymph node to facilitate their differentiation [11]. This phenomenon of S1P_1_‐mediated lymphocyte retention in lymph nodes has been exploited to develop a class of small molecule drugs, known as S1PR modulators, for treatment of multiple immune‐mediated inflammatory diseases via the activation of intracellular G protein and β‐arrestin signaling cascades [12, 13, 14]. S1PR modulators induce sustained S1P_1_ internalization and proteasomal/lysosomal degradation resulting in loss of the cell surface‐expressed receptor [9]. Due to the loss of surface receptor, S1P can no longer signal through S1P_1_ and mediate lymphocyte trafficking; therefore, these S1PR modulators act as functional antagonists, retaining lymphocytes within lymph tissue [15].

In addition to the effects on lymphocyte circulation, activation of G proteins by S1PR modulators can lead to a variety of downstream functional outcomes, including transient heart rate reduction [16]. S1P_1_‐mediated G protein activation triggers G protein‐coupled inwardly rectifying potassium (GIRK) channels on the surface of cardiomyocytes within the sinoatrial and atrioventricular nodes, prolonging repolarization and causing heart rate reduction and conduction delays [12, 13, 14]. This effect is transient in nature as ongoing β‐arrestin‐mediated receptor internalization and degradation deplete the available pool of cell surface receptor, attenuating the effects of subsequent doses on heart rate [3, 17]. Due to on‐target activation of GIRK channels, S1PR modulators often necessitate drug up‐titration over several days to help mitigate this cardiac effect [18]. Based on these observations and the known concept of biased receptor signaling, S1P_1_ ligands that preferentially activate β‐arrestin over the G protein pathways could sustain a potent effect on lymphocyte trafficking with decreased impact on first‐dose heart rate reduction [19, 20].

Currently, all clinically tested and approved S1PR modulators activate one or more S1PR. Given the versatile and nonredundant roles fulfilled by various members of the S1PR family, target specificity of S1PR modulators is thought to have a meaningful impact on their therapeutical efficacy and safety profiles. The first S1PR modulator to obtain marketing authorization, fingolimod (FTY720), is a potent agonist of S1P_1_, S1P_3_, S1P_4_, and S1P_5_ [21]. This nonselectivity has been hypothesized to contribute to the frequency and variety of observed adverse events, such as macular edema, decreased pulmonary function, elevated liver enzymes, and neoplasms [22, 23, 24]. Many second‐generation S1PR modulators, including siponimod [25] and ozanimod [12], were designed to be more S1P_1_ specific, largely devoid of activity at S1P_2_ and S1P_3_, and are reported to be targeted agonists of S1P_1_ and S1P_5_.

Etrasimod is an oral, once‐daily, selective sphingosine 1‐phosphate (S1P)_1,4,5_ receptor modulator for the treatment of moderately to severely active ulcerative colitis (UC). The efficacy and safety of etrasimod are also under investigation in other immune‐mediated inflammatory diseases, including atopic dermatitis (ADVISE, NCT04162769 [26]), Crohn's disease (CULTIVATE, NCT04173273), eosinophilic esophagitis (VOYAGE, NCT04682639 [27, 28]), and alopecia areata (NCT04556734). In patients with moderately to severely active UC, the phase III ELEVATE UC 52 (NCT03945188) and ELEVATE UC 12 (NCT03996369) trials of etrasimod demonstrated statistically significant improvements in all primary and secondary endpoints at Weeks 12 and 52, when compared to placebo [29]. While individual reports of pharmacological properties of etrasimod, fingolimod, siponimod, and ozanimod have been previously published [30, 31, 32, 33], different methods and assay conditions make individual potency and efficacy values hard to compare directly. Here, we pharmacologically profiled etrasimod comparing it directly to fingolimod phosphate, siponimod, ozanimod, and its predominant metabolite (ozanimod ketone or CC112273).

## Materials and methods

### Cell culture

Recombinant Chinese Hamster Ovary K1 (CHO‐K1) cell lines (Beacon Discovery, San Diego, CA, USA) were cultured in F12 media supplemented with 10% fetal bovine serum (FBS), 100 mg·mL^−1^ penicillin, 100 U·mL^−1^ streptomycin, and 500 mg·mL^−1^ G418 at 37 °C and 5% CO_2_. Human umbilical vein endothelial cells (HUVECs) were obtained from Cell Applications (San Diego, CA, USA) and were cultured in endothelial cell growth media (ECGM) from the same supplier in a humidified incubator at 37 °C and 5% CO_2_.

### Test compounds

S1P, the active metabolite of fingolimod (fingolimod phosphate [fingolimod(P)]), ozanimod, and siponimod were purchased from Cayman Chemical (Ann Arbor, MI, USA). Etrasimod and ozanimod ketone (ozanimod[K]) were synthesized by Arena Pharmaceuticals, Inc. (San Diego, CA, USA). Stock solutions of etrasimod, ozanimod, ozanimod(K), and siponimod were prepared at concentrations of 20 mm in dimethyl sulfoxide (DMSO). Fingolimod(P) stock solution was prepared at a concentration of 5 mm in DMSO. Serial dilutions were prepared in DMSO at 200× the final assay concentration along a 10‐point dose–response curve, serially diluted 1 : 5 from the highest final concentration of 10 μm. S1P stock solution was prepared at a concentration of 2 mm in water containing 4 mg·mL^−1^ fatty acid‐free bovine serum albumin (BSA).

### β‐Arrestin recruitment assay

β‐Arrestin recruitment assays [34] were performed using the PathHunter β‐arrestin assay platform from DiscoverX (Fremont, CA, USA), according to the manufacturer's instructions. Briefly, PathHunter HEK293 cells stably expressing ProLink‐tagged S1PRs were plated at a density of 5000 cells per well in 8 μL of Opti‐MEM media (Gibco/Thermo Fisher, Waltham, MA, USA) containing 0.1% fatty acid‐free BSA (Roche, Indianapolis, IN, USA) in 384‐well ProxiPlates (Perkin Elmer, Waltham, MA, USA) and incubated overnight in a humidified incubator at 37 °C and 5% CO_2_. On the next day, plates were removed from the incubator and equilibrated to room temperature for 1 h prior to addition of test compounds. Test compounds were serially diluted in Opti‐MEM containing 0.1% fatty acid‐free BSA at 5× the final assay concentrations. Next, 2 μL of test compound solutions were added to cells, and assay plates were incubated at room temperature for 2 h. Lysis/detection reagents (8 μL per well) were then added, and plates were sealed and incubated for an additional 4 h at room temperature. Plates were read on an EnVision plate reader (PerkinElmer).

### GTPγS binding assay

To prepare membranes for GTPγS binding assays, CHO‐K1 cells stably expressing human S1PRs were grown in 5‐layer cell culture flasks. Cells were washed with 50 mL of phosphate‐buffered saline (PBS) and then dissociated with 50 mL of cell dissociation buffer (5 mm HEPES, 10 mm EDTA, 2 mm EGTA, pH 7.4). Dissociated cells were then collected by centrifugation at 300 ***g*** for 5 min. Pellets were resuspended in 10 mL of wash buffer (20 mm HEPES, 10 mm EDTA, pH 7.4) followed by centrifugation at 50 000 ***g*** for 15 min at 4 °C. Membrane pellets were resuspended in 5 mL of storage buffer (20 mm HEPES, pH 7.4) followed by centrifugation at 50 000 ***g*** for 15 min at 4 °C. Supernatants were removed and pellets were stored at −80 °C until the assay. On the assay day, pellets were thawed on ice and resuspended in 1 mL of 20 mm HEPES, pH 7.4, 10 mm MgCl_2_, 200 mm NaCl using a Dounce homogenizer. Membrane protein concentration was determined by BCA protein assay (Promega, Madison, WI, USA).

For the assay, 50 μL per well of membrane homogenates containing 5 to 10 μg of membrane protein in assay buffer (20 mm HEPES, 10 mm MgCl_2_, 200 mm NaCl, 10 mm GDP, 0.1% fatty acid‐free BSA) were dispensed into 96‐well Scintiplates (Perkin Elmer). Then, 25 μL of test compounds serially diluted in assay buffer at 4× the final assay concentration were added to membrane homogenates, and plates were incubated for 5 min at room temperature with gentle shaking before the addition of 25 μL of 0.4 nm ^35^S‐GTP (Perkin Elmer) in assay buffer. The binding reaction was incubated on a shaker for 1 h at room temperature before plates were centrifuged at 3200 ***g*** for 15 min. Supernatants were aspirated with a manifold, and plates were then sealed and read on a TopCount scintillation counter (Perkin Elmer).

### cAMP assay

The cellular cAMP levels were measured using a 384‐well homogeneous time‐resolved fluorescence assay (HTRF cAMP dynamic 2 assay, Cisbio International, Bagnols‐sur‐Cèze, France) following the manufacturer's instructions. Recombinant CHO‐K1 cells and HUVECs resuspended in PBS supplemented with 0.1% fatty acid‐free BSA and 500 mm IBMX were dispensed (5 μL per well) at 2000 and 1000 cells per well, respectively, into 384‐well ProxiPlates (Perkin Elmer). Test compounds were serially diluted at 2× the final assay concentration in PBS with 0.1% fatty acid‐free BSA, 10 μm forskolin, and 500 mm IBMX. Then, 5 μL per well of test compound stocks were added to the cell plates, and plates were incubated for 1 h at room temperature. The stimulation was terminated by sequential addition of 5 μL per well cAMP‐d2 and 5 μL per well anti‐cAMP cryptate conjugate, both diluted as recommended in a lysis and detection buffer supplied with the assay kit. Plates were incubated for 1 h at room temperature and read on an EnVision plate reader (PerkinElmer).

### S1P_1_ internalization assay

S1P_1_ internalization was measured in CHO‐K1 cells stably expressing recombinant S1P_1_ tagged with hemagglutinin (HA). Prior to the assay, cultured cells were serum starved for 24 h in F12 media. Cells were harvested using Cell Dissociation Buffer (Gibco/Thermo Fisher), washed in PBS, and resuspended in F12 media supplemented with 0.1% fatty acid‐free BSA. Cells were dispensed into 96‐well plates (350 k cells per well in 45 μL), followed by the addition of 15 μL per well of test compounds serially diluted at 4× the final assay concentration in F12 media supplemented with 0.1% fatty acid‐free BSA. Plates were incubated for the indicated time period in a shallow water bath in a tissue culture incubator at 37 °C and 5% CO_2_. After incubation, ice‐cold PBS was added to the wells (200 μL per well), and plates were incubated on ice for 10 min. Cells were collected by centrifugation (300 ***g***, 5 min) at 4 °C and washed twice by centrifugation with 200 mL of ice‐cold PBS. Cell surface S1P_1_ was labeled with 2 μg·mL^−1^ of Alexa Fluor 488‐conjugated anti‐HA epitope antibody (R&D Systems, Minneapolis, MN, USA) in PBS supplemented with 1% FBS. Cells were incubated with the antibody for 1 h on ice, washed twice with ice‐cold PBS, and resuspended in ISOTON II diluent (Beckman Coulter, Brea, CA, USA). Flow cytometry was performed on an LSR II (BD Bioscience, San Jose, CA, USA), and surface‐associated Alexa Fluor 488 label was quantified with facs diva.

### Immunofluorescent microscopy

PathHunter HEK293 cells stably expressing S1P_2_ with an N‐terminal HA tag were plated (50 000 cells per well) in an 8‐well poly‐D‐lysine (Sigma‐Aldrich, St. Louis, MO, USA)‐coated glass chamber slides (Thermo Fisher) in normal cell culture media with 10% FBS and incubated for 18 h in a humidified incubator at 37 °C and 5% CO_2_. Cells were washed twice with PBS and incubated in serum‐free Opti‐MEM media for 2 h. Alexa Fluor 488‐conjugated anti‐HA monoclonal antibody (R&D Systems) was added to the cells at a final concentration of 2 μg·mL^−1^, and cells were incubated for 20 min at room temperature. Vehicle (0.5% DMSO in Opti‐MEM media) or test compounds (5 μm) were then added, and cells were incubated for 90 min in a tissue culture incubator at 37 °C and 5% CO_2_. At the end of the 90‐min incubation, rhodamine‐conjugated transferrin was added to all wells at a final concentration of 30 μg·mL^−1^, and cells were incubated for an additional 30 min in a tissue culture incubator. Cells were washed twice with PBS and fixed in 4% paraformaldehyde (Sigma‐Aldrich) in PBS for 20 min at 4 °C. After washing with PBS, the chamber system was disassembled, and the glass slide was extracted. A glass coverslip was mounted on the slide using the Vecta‐shield anti‐fade kit (Cole‐Parmer, Vernon Hills, IL, USA). Images were collected using a zeiss axio observer.z1 fluorescent microscope.

### Calculation of signaling bias

Differential signaling between compounds was assessed by calculation of signaling bias factors using a relative activity ratio‐based method, as described previously [35, 36]. In all S1P_1_ *in vitro* signaling assays, S1P was used as the reference compound for normalization. First, calculations were performed to determine Δlog(*E*_max_/EC_50_) in a specific signaling pathway, as follows:

$$\text{Δlog}\left(\frac{E_{\max}}{{\mathrm{EC}}_{50}}\right)=\log\left(\frac{E_{\max}B}{{\mathrm{EC}}_{50}B}\right)-\log\left(\frac{E_{\max}A}{{\mathrm{EC}}_{50}A}\right)$$

where A and B are the reference and test compound, respectively. Only curves where reference compound S1P was dosed along a test compound were used for these calculations. The Δlog(*E*_max_/EC_50_) values were calculated within each individual determination, and then the values were averaged to calculate a mean Δlog(*E*_max_/EC_50_). For estimation of signaling bias between pathways, pathway bias factors ΔΔlog(*E*_max_/EC_50_) were calculated as follows:

$$\Delta \Delta \log\left(E_{\max}/{\mathrm{EC}}_{50}\right)=\begin{array}{l} \Delta \log{\left(E_{\max}/{\mathrm{EC}}_{50}\right)}_{\text{pathway}\;1}\\ -\Delta \log{\left(E_{\max}/{\mathrm{EC}}_{50}\right)}_{\text{pathway}\;2} \end{array}$$

### GIRK channel activation

All human hearts used for this study were nontransplantable and ethically obtained by legal written informed consent (first person or next of kin) from cadaveric organ donors in the United States. Recovery protocols and *in vitro* experimentation were preapproved by Institutional Review Boards at transplant centers within the US Organ Procurement Transplant Network. Furthermore, all transfers of the donor hearts are fully traceable and periodically reviewed by US federal authorities. Donor characteristics, heart number, and donor identifier are shown in Table S1, and exclusion criteria were previously described [37, 38, 39].

Upon arrival, hearts were reperfused with ice‐cold proprietary cardioplegic solution, and adult human primary atrial myocytes were isolated enzymatically from the atria [37, 39]. The whole‐cell configuration of the patch‐clamp technique was used to record GIRK current. The cells were held in the voltage‐clamp mode using an EPC‐10 USB amplifier (HEKA Elektronik, Reutlingen, Germany). Myocytes were held at a potential of −70 mV for 100 ms, then a ramp pulse was applied from +45 mV to −140 mV for 1 s. This ramp pulse was followed by a pulse back to the holding potential for 100 ms. Interstep intervals for recordings of GIRK current were of 2 s. GIRK current was reported as peak current amplitude between −90 and −140 mV. Series resistance was compensated at 50%, and current measurements were normalized to cell capacitance to estimate GIRK current density. Patch pipettes were pulled from filamented borosilicate glass (Warner Instruments, Holliston, MA, USA) using a P‐97 puller (Sutter Instruments, Novato, CA, USA) and had tip resistance of approximately 4 MΩ when filled with the internal solution. All recordings were carried out at approximately 37 °C, and signals were acquired at a sampling rate of 10 kHz, filtered at 1 KHz and analyzed offline using the fitmaster analysis software package (HEKA Elektronik). The composition of the internal solution was as follows (in mm): 110 K^+^ aspartate, 20 KCl, 10 EGTA, 8 NaCl, 4 K_2_ ATP, 1 MgCl_2_, 1 CaCl_2_, 0.01 GTP Tris, and 10 HEPES; pH adjusted to 7.3 with KOH and osmolarity to 280 mOsm. The composition of the external solution was as follows (in mm): 140 NaCl, 5.4 KCl, 1 MgCl_2_, 1 CaCl_2_, 5.5 Dextrose, 10 HEPES, and 0.33 Na_2_HPO_4_; pH adjusted to 7.4 using NaOH and osmolarity to 300 mOsm. Three ascending concentrations of S1PR modulators were used, and each concentration was perfused for 5 min. 10 μm Carbachol (CCh), an activator of the GIRK channel, was used at the end of each experiment as the positive control.

### Statistical analysis

Dose–response curves were fit to a nonlinear least‐squares curve‐fitting program in graphpad prism 5 (GraphPad Software, La Jolla, CA, USA) to obtain EC_50_ and IC_50_ values. All data are reported as means ± SD. Statistical significance was determined using unpaired *t*‐tests or two‐way analysis of variance.

## Results

### Comparative intracellular signaling of S1PR modulators at S1P_1_

**Fig. 2:**
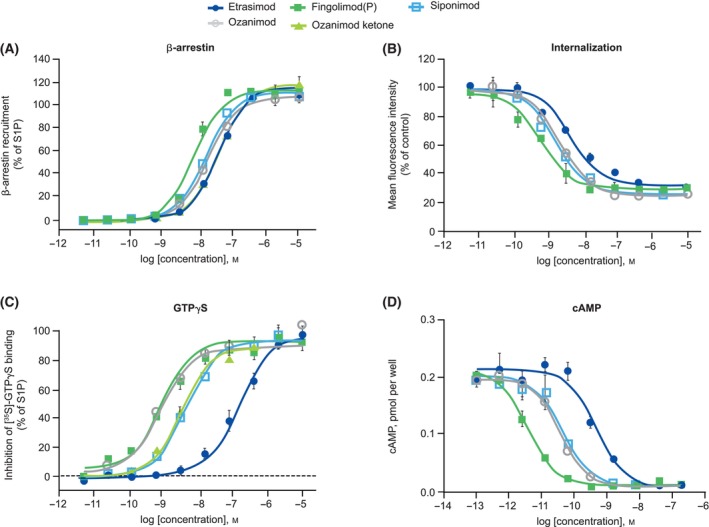
Concentration–response curves in cell lines stably transduced to express hS1P_1_ for (A) β‐arrestin recruitment, (B) receptor internalization, (C) GTPγS binding, and (D) cAMP inhibition. All assays were performed in media with identical protein concentration (0.1% BSA). Error bars represent standard deviation. Panels (A–D) present technical replicates. (A) and (D): *n* = 4; (B) and (C): *n* = 3. BSA, bovine serum albumin; *n*, number of tests; S1P, sphingosine 1‐phosphate.

Using CHO‐K1 cells stably transduced with hS1P_1_, potencies (EC_50_) and efficacies (*E*_max_) for β‐arrestin signaling were calculated for etrasimod, fingolimod(P), ozanimod, ozanimod(K), and siponimod, and were directly compared with S1P. All tested compounds proved to be full S1P_1_ agonists with similar *E*_max_ values and demonstrated similar EC_50_ values of 44, 33.2, 73.9, 11, 53.9, and 21 nm for etrasimod, ozanimod, ozanimod(K), Fingolimod(P), siponimod, and S1P, respectively (Fig. 2A; Table 1).

**Table 1:** Summary of pharmacologic profiling. CI, confidence interval; EC_50_, concentration required for 50% of the maximum effect; *n*, number of tests; NR, no response; S1P, sphingosine 1‐phosphate.

Assay	Etrasimod	Ozanimod	Ozanimod(K)	Fingolimod(P)	Siponimod	S1P
β‐Arrestin						
EC_50_, mean (95% CI) [*n*], nm	44 (38.3–50.5) [3]	33.2 (19.8–55.7) [4]	73.9 (43.8–124) [4]	11 (7.9–15.3) [4]	53.9 (12.7–229) [4]	21 (9.7–45.6) [4]
*E*_max_, % of S1P	120	114	123	109	111	111
Internalization						
EC_50_, mean (95% CI) [*n*], nm	8.3 (4.1–16.6) [4]	3.5 (2.1–5.6) [8]	NR	1.4 (0.3–6.8) [4]	1.8 (0.9–3.9) [4]	608.1 (35.4–10 447) [4]
*E*_max_, % of S1P	106.9	121.6	NR	112	113.3	111.2
GTPγS						
EC_50_, mean (95% CI) [*n*], nm	57 (18–180) [4]	1.2 (0.7–2) [4]	7.3 (2.7–19.9) [4]	1.3 (0.4–3.6) [4]	3.7 (1.8–7.7) [4]	156 (108–226) [4]
*E*_max_, % of S1P	99	103	97	86	93	101
cAMP						
EC_50_, mean (95% CI) [*n*], nm	0.465 (0.336–0.643) [13]	0.039 (0.030–0.052) [13]	NR	0.008 (0.005–0.012) [11]	0.028 (0.017–0.047) [13]	0.028 (0.019–0.039) [12]
*E*_max_, % of S1P	103.3	99.8	NR	105.8	105.1	103.6

All tested S1PR modulators induced robust S1P_1_ internalization in transduced CHO‐K1 cells with *E*_max_ values closely matching that of S1P. They also shared similar potencies of 8.3 nm for etrasimod, 3.5 nm for ozanimod, 1.4 nm for fingolimod(P), and 1.8 nm for siponimod that were all significantly higher than that of S1P (608.1 nm) (Fig. 2B; Table 1). Collectively, these data indicate that etrasimod, fingolimod(P), ozanimod, and siponimod have similar pharmacologic properties, in terms of inducing β‐arrestin recruitment and driving S1P_1_ internalization.

All tested compounds behaved as full agonists in the GTPγS binding assay and the cAMP assay on CHO‐K1 cells transduced with S1P_1_ (Fig. 2C,D; Table 1), but with a range of potencies—etrasimod being the least potent among all tested synthetic S1PR modulators. Notably, potencies of all tested compounds were significantly higher in the cAMP inhibition assay than in the GTPγS binding assay. The relative differences, or rank order in potency between etrasimod, ozanimod, fingolimod(P), and siponimod were similar to those observed in the GTPγS binding tests.

**Fig. 3:**
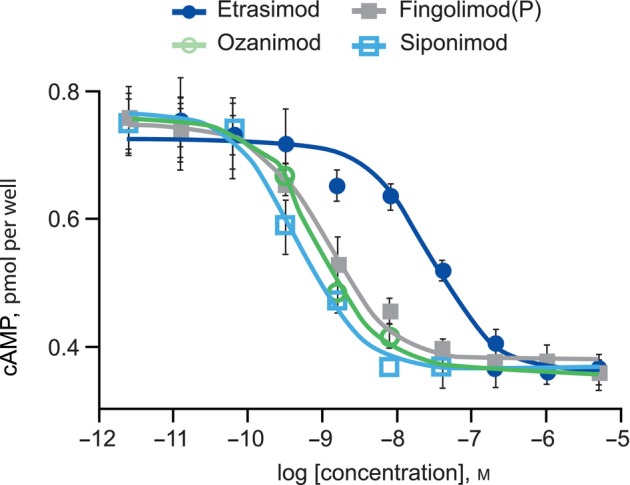
Concentration–response curves for cAMP assay using HUVECs known to naturally express S1P_1_. Error bars represent standard deviation (*n* = 4). This figure presents technical replicates. HUVEC, human umbilical vein endothelial cells; *n*, number of tests; S1P, sphingosine 1‐phosphate.

Repeated testing using HUVECs, which naturally express S1P_1_, confirmed cAMP potency and efficacy observations (Fig. 3; Table 2). Ozanimod, fingolimod(P), and siponimod all inhibited cAMP production in HUVECs with similar potencies (EC_50_ of 2, 1.6, and 1.6 nm, respectively), whereas etrasimod was less potent with an EC_50_ of 30 nm. Collectively, these data suggest in both recombinant and endogenously expressing cells that etrasimod has a weaker propensity than other S1PR modulators to initiate S1P_1_/G_i_ protein signaling and subsequent inhibition of cAMP synthesis.

**Table 2:** Pharmacologic profiling for cAMP assay using HUVECs known to naturally express S1P_1_. CI, confidence interval; EC_50_, concentration required for 50% of the maximum effect; HUVEC, human umbilical vein endothelial cells; *n*, number of tests; NR, no response; S1P, sphingosine 1‐phosphate.

Assay	Etrasimod	Ozanimod	Ozanimod(K)	Fingolimod(P)	Siponimod	S1P
cAMP						
EC_50_, mean (95% CI) [*n*], nm	30.5 (19.9–46.7) [7]	2.0 (1.2–3.1) [11]	NR	1.6 (1.2–2.3) [6]	1.6 (1–2.4) [9]	19.1 (9.5–38) [8]
*E*_max_, % of S1P	105.2	108.3	NR	98.4	106.1	98.6

### Signaling bias of S1PR modulators

**Fig. 4:**
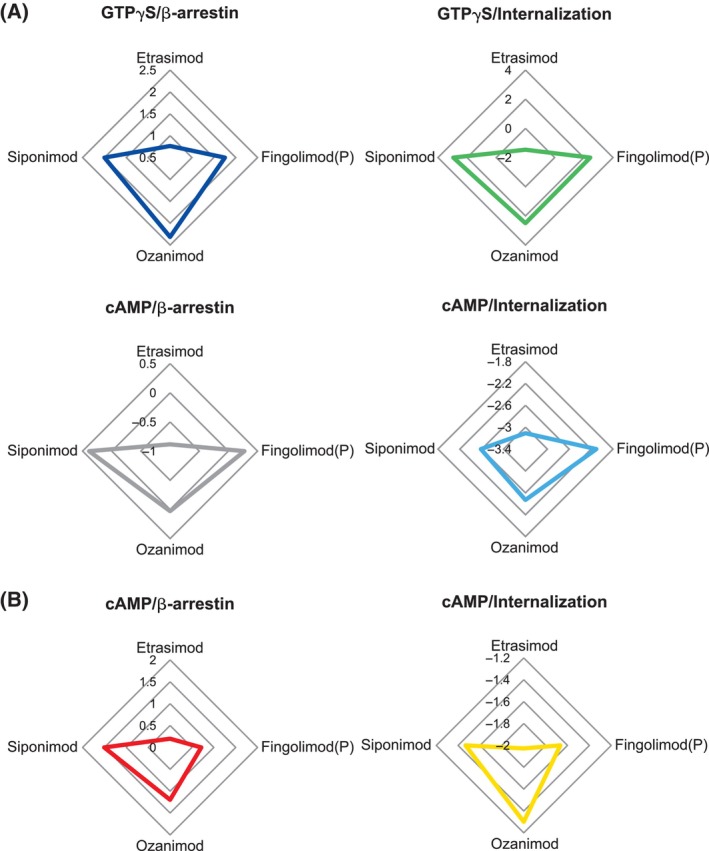
Calculated relative bias of S1PR modulators in activating G protein‐dependent signaling (GTPγS and cAMP) versus G protein‐independent signaling (β‐arrestin and receptor internalization). The relative bias factors were calculated using the formula ΔΔlog(*E*_max_/EC_50_) and normalized to the bias of S1P that was given as 0. Assays presented in plot group (A) were performed using cell lines stably transduced with hS1P_1_. In plot group (B), the cAMP assay was performed using HUVEC cells naturally expressing S1P_1_. The values represent the fold change relative to the bias calculated for S1P and given as 0. EC_50_, concentration required for 50% of the maximum effect; HUVEC, human umbilical vein endothelial cells; S1P, sphingosine 1‐phosphate; S1PR, sphingosine 1‐phosphate receptor.

To quantitatively compare the differences between etrasimod and other tested S1PR modulators, we calculated bias factors characterizing the potency of initiating G protein‐dependent signaling versus β‐arrestin/internalization pathways. Since various signaling pathways were interrogated through separate cellular assays, the calculated biases should be seen as relative deviation from the signaling balance produced by stimulation with S1P. The logarithmic character of the calculated bias means that a value of 0 represents no bias relative to S1P, whereas a value of 1 represents a 10‐fold difference in preference to activate one of the compared pathways. When compared to other S1PR modulators, etrasimod was generally less biased toward initiating G protein‐dependent signaling measured by GTPγS binding and inhibition of cAMP synthesis (Fig. 4; Table 3).

**Table 3:** Calculated relative bias factors for S1P_1_ in activating G protein‐dependent signaling (GTPγS and cAMP) versus G protein‐independent signaling (β‐arrestin and receptor internalization). The relative bias factors were calculated using the formula ΔΔLog(*E*_max_/EC_50_). The values represent the fold change relative to the bias calculated for S1P and given as 0. EC_50_, concentration required for 50% of the maximum effect; HUVEC, human umbilical vein endothelial cells; S1P, sphingosine 1‐phosphate.

Compared assays	Etrasimod	Ozanimod	Fingolimod(P)	Siponimod	S1P
GTPγS/β‐arrestin	0.76	2.31	1.75	2.0	0
GTPγS/internalization	−1.46	2.5	2.44	2.95	0
cAMP/Internalization	−3.11	−2.47	−2.10	−2.59	0
cAMP/β‐arrestin	−0.88	0.03	0.27	0.39	0
HUVEC cAMP/β‐arrestin	0.2	1.2	0.71	1.51	0
HUVEC cAMP/internalization	−2.03	−1.30	−1.67	−1.47	0

### Impact of S1PR modulators on GIRK channel activity

**Fig. 5:**
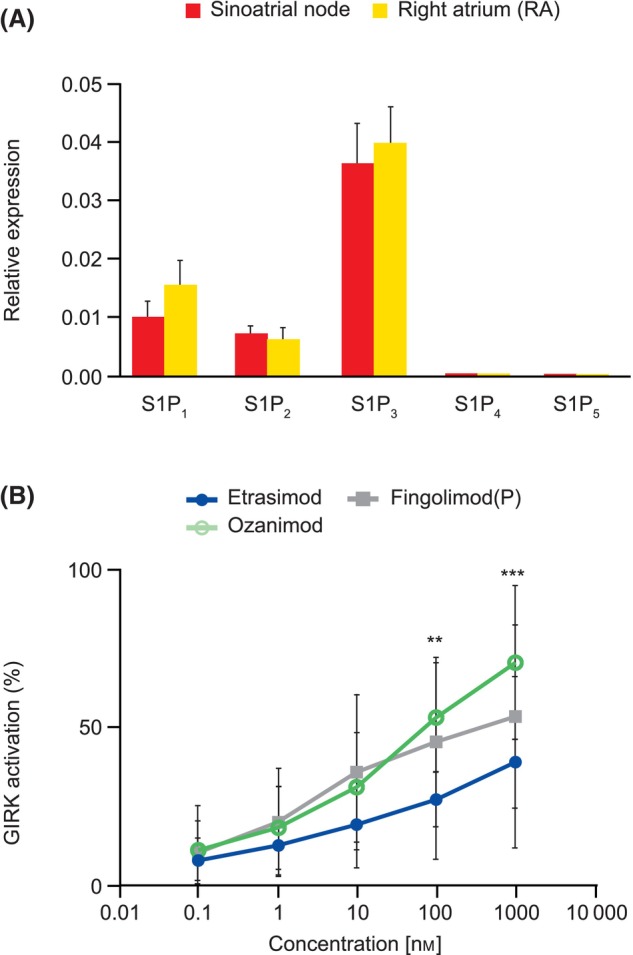
Effect of S1P receptor modulators on cardiac tissue. (A) mRNA expression levels of S1PR modulators in human cardiac tissues from the right atrium and sinoatrial node normalized to human Ribosomal Protein S9. Data represent mean values measured for four donors. Error bars represent standard deviation. (B) Activity of etrasimod, ozanimod, and fingolimod(P) in promoting GIRK channel activity in human atrial cardiomyocytes. Data represent mean values measured for six donors. Error bars represent standard deviation. Two‐way ANOVA was used to compare the activity of etrasimod and ozanimod (**P = 0.0057, ***P = 0.0009). Corresponding values for comparing the activity of etrasimod and fingolimod(P) were *P* = 0.1097 and *P* = 0.3275, respectively. ANOVA, analysis of variance; GIRK, G protein‐coupled inwardly rectifying potassium; S1P, sphingosine 1‐phosphate; S1PR, sphingosine 1‐phosphate receptor.

To test whether the low potency of etrasimod for G protein activation among S1PR modulators translates into lower potential for GIRK channel stimulation, we measured GIRK activation in human cardiomyocytes. First, we assessed the levels of mRNA expression for all five S1P receptors in cardiac tissue isolated from the sinoatrial node (SAN) and right atria (RA) of human donor hearts. Both SAN and RA had very similar expression profile characterized by the highest expression of S1P_3_, expression of S1P_1_ and S1P_2_ was about 3–4 times lower than S1P_3_, and levels of S1P_4_ and S1P_5_ levels were detected only at very low levels (about 100 times less than S1P_3_) (Fig. 5A). Given this similar expression pattern in both cell types, we used RA myocytes in further tests based on better availability of these cells. All tested compounds stimulated GIRK channels in a dose‐dependent manner (Fig. 5B). Etrasimod was shown to be a less effective GIRK activator than ozanimod, the difference reaching statistical significance at concentrations of 100 and 1000 nm, while fingolimod(P) trended toward higher GIRK channel activation compared with etrasimod.

### Pharmacological properties of S1PR modulators at S1P_2_ _–5_

We extended our pharmacological analysis of etrasimod and the other test compounds on S1P_2–5_. Etrasimod demonstrated no response at S1P_2_ up to maximal tested concentration of 10 μm (Table S2). Similarly, ozanimod, its ketone metabolite, and siponimod showed no agonist activity at S1P_2_ in the β‐arrestin and G protein assays, and fingolimod(P) was a weak agonist, in terms of both potency and efficacy.

S1P_2_ internalization in response to test compounds was measured via fluorescence microscopy (Fig. S1A). Fingolimod(P) and ozanimod induced robust S1P_2_ internalization in a dose‐dependent and saturable fashion (Fig. S1B), while etrasimod was completely inactive in inducing S1P_2_ internalization over the tested dose range. Further studies showed that this S1P_2_ internalization was mediated by an arrestin‐independent, dynamin‐dependent, clathrin‐mediated endocytic pathway (data not shown).

Although etrasimod was devoid of any agonism on S1P_3_ in terms of activating the β‐arrestin recruitment pathway, it showed some weak agonism in the GTPγS binding assay with EC_50_ of 6050 nm, well in excess of a therapeutic concentration, and an efficacy of 41.5% compared to S1P (Table S2). From all synthetic compounds that were tested, only fingolimod(P) was found to be a partial but potent agonist in terms of G protein activation (EC_50_ of 13.8 nm), and β‐arrestin recruitment (22.3 nm). Similar to etrasimod, ozanimod showed low potency with EC_50_ of > 6000 nm and *E*_max_ of ~ 50% in the GTPγS binding assay. Neither ozanimod(K) nor siponimod activated G protein signaling via S1P_3_ in the tested concentration range of up to 10 μm.

All tested compounds showed agonist activity in the β‐arrestin recruitment assay on cells stably expressing S1P_4_ (Table S2). Etrasimod was a partial agonist with EC_50_ of 141 nm and *E*_max_ of 53.9%, relative to S1P. Fingolimod(P) proved to be a much more potent and efficacious compound, in the context of S1P_4_ agonism, with EC_50_ of 6.9 nm and Emax of 77.6%. In turn, ozanimod, its metabolite, and siponimod had EC_50_ values exceeding 500 nm. In the GTPγS binding assay, only fingolimod(P) and siponimod exhibited agonist activity at S1P_4_ (EC_50_ of 6.1 and 510 nM, respectively), while etrasimod, ozanimod, and ozanimod(K) were inactive in this assay at concentrations up to 10 μm.

All tested compounds were agonists of S1P_5_, inducing both G protein signaling and β‐arrestin recruitment (Table S2). The most potent were fingolimod(P) (EC_50_ of 5.8 and 1.07 nm respectively) and siponimod (11 and 0.54 nm). Etrasimod (63.4 and 63.5 nm), ozanimod (28.8 and 12.3 nm), and ozanimod(K) (58.5 and 95.8 nm) were less potent. Potencies for S1P in these two assays were 1.4 and 49.7 nm, respectively. In terms of efficacy relative to S1P, ozanimod, its metabolite, and siponimod behaved as full agonists, whereas etrasimod had *E*_max_ values of 85.7% in the β‐arrestin recruitment assay and 72% in the GTPγS binding assay. Similarly, the *E*_max_ values for fingolimod in these assays were 48.4% and 69.5%, respectively.

## Discussion

This is one of the first analyses to assess the pharmacologic profile of multiple S1PR modulators in a head‐to‐head setting. Using identical experimental conditions, we directly compared the relative activity, selectivity, and bias of etrasimod with other approved S1PR modulators fingolimod, ozanimod, and siponimod. Etrasimod demonstrated balanced signaling through β‐arrestin and G protein‐related pathways at S1P_1_, did not engage S1P_2_ and S1P_3_ at therapeutic concentrations, and was an activator of S1P_4_ and S1P_5_ β‐arrestin pathways. All tested compounds shared a similar potency of activating the S1P_1_ β‐arrestin pathway but differed in their capacities to activate G protein‐related pathways and downstream GIRK activation through this receptor.

S1PR modulators are reported to project their therapeutic effect by driving functional antagonism of S1P_1_ [15] through β‐arrestin signaling. We measured the *in vitro* potencies of etrasimod and other tested compounds at inducing β‐arrestin recruitment and receptor internalization, and found that all compounds were potent full agonists of S1P_1_ in these assays. Our results are consistent with the outcomes of S1PR modulator clinical trials, which demonstrate reductions in circulating lymphocytes driven by the functional antagonism of S1P_1_ [22, 29, 40].

In contrast, the same S1PR modulators showed different potencies at inducing G protein activation through S1P_1_ in both heterologous expression systems and primary cells. Of all tested compounds, etrasimod was the least potent in both the GTPγS and cAMP inhibition assays used to measure activation and downstream effects of G_i_ activation, respectively. Using a standard calculation of receptor signaling bias [35], we determined that, among the tested S1PR modulators, etrasimod was the least G protein biased when activating S1P_1_. The S1P_1_‐mediated activation of G_i_ protein signaling is thought to be responsible for the transient first‐dose heart rate reduction [41].

We speculated that the lower S1P_1_‐induced G protein signaling following treatment with etrasimod, as compared to other S1PR modulators, would result in reduced GIRK activation, and translate to less impact on heart rate upon first dose. Our results suggest that at equivalent concentrations, etrasimod was a less potent activator of the GIRK channel compared with ozanimod, and that etrasimod may have a lower intrinsic propensity to induce first‐dose heart rate reductions at therapeutic doses with similar degrees of lymphocyte reduction. Phase III trials of etrasimod demonstrated a low incidence of cardiac‐related adverse events with no dose titration, consistent with the receptor signaling bias and downstream channel activation demonstrated within this study [42]. However, several other factors could influence the overall effect of S1PR modulators on heart rate reduction after first dose, including the rate of absorption, volume of distribution, and metabolism. Previous analyses have shown that S1P_1_ is the primary receptor subtype expressed by cardiac myocytes, the activation of which inhibits cAMP formation and antagonizes adrenergic receptor‐mediated contractibility; S1PR activation has been linked to cardioprotection, hypertrophy induction, and modulation of intracellular calcium [43, 44, 45, 46, 47, 48, 49, 50]. Analysis of cAMP inhibition and GTPγS binding were unable to be performed in our analysis due to the fragility and poor long‐term viability of human RA myocytes in culture.

In addition to the unique properties of etrasimod with respect to S1P_1_ activation, we have also demonstrated its distinctive selectivity profile across the other four S1P receptors, S1P_2–5_. Our results demonstrated that all tested S1PR modulators had negligible activity at S1P_2_, consistent with advanced generations of S1PR modulators being designed to be more selective and generally devoid of S1P_2_ activity [51]. Notably, while ozanimod induced robust internalization of S1P_2_, our analysis did not show a downstream G‐protein signal. It is therefore unclear whether this S1P_2_ internalization is physiologically relevant. At S1P_3_, only fingolimod(P) was an S1P_3_ agonist, which is consistent with published reports [52] and may contribute to reported incidences of adverse events, such as increased blood pressure, decreased pulmonary function, and macular edema [22, 23, 24]. These adverse events are significantly less common in patients treated with more selective S1PR modulators lacking S1P_3_ activity, such as ozanimod or etrasimod [53, 54, 55, 56]. Among the tested S1PR modulators, only fingolimod(P) was a full and potent agonist and activated both G protein‐ and β‐arrestin‐mediated pathways through S1P_4_. In contrast, etrasimod was a partial S1P_4_ agonist, which indicates that this activity could potentially have an impact on the function of immune cells that largely express S1P_4_, such as lymphocytes, neutrophils, macrophages, and dendritic cells [5]. In our study, all of the characterized S1PR modulators were potent activators of both G protein‐ and β‐arrestin‐mediated pathways through S1P_5_. Among immune cells, S1P_5_ expression is largely confined to NK cells, T cells, oligodendrocytes, and keratinocytes [5, 6, 7, 8], and could potentially be involved in the trafficking of NK cells and their mobilization to inflamed organs [6, 57, 58], which may contribute to the therapeutic benefits seen across S1PR modulators.

Our study results should be interpreted in the context of some limitations. Signaling bias studies were performed using heterologous expression systems, artificial recombinant systems, and primary cells, and may not fully represent S1PR modulator activities in other tissues or in physiological settings. Although significant advancements in the biological activity of S1P_1–5_ have been published, studies examining the direct role of S1PR biological functions are still ongoing. While it is tempting to speculate that these unique pharmacological properties of etrasimod could translate into improved benefit–risk profile, the clinical relevance of these findings has yet to be elucidated and only robust, preferably head‐to‐head, clinical studies would be able to shed light on this question.

In summary, our direct comparison of etrasimod with other clinical S1PR modulators revealed that they all share similar potency of activating the β‐arrestin pathway through S1P_1_, but differ in their capacities to activate G protein‐related pathways and promote downstream GIRK activation through this receptor, which is thought to be related to first‐dose heart rhythm effects. Like other second‐generation S1PR modulators, etrasimod did not engage S1P_2_ and S1P_3_ at therapeutic concentrations but was a partial agonist of the β‐arrestin pathways via S1P_4_ and S1P_5_.

## Conflict of interest

IG, KB, HD, XC, TA, JG, DU, and HA‐S were employees of Beacon Discovery at the time of completion of work; HKK, DTS, LK‐N, CJR, CC, and JWA were employees of Arena Pharmaceuticals, a wholly owned subsidiary of Pfizer, Inc, at the time of completion of work; A‐TT, TI, PEM, AG, and NA‐G were employees of AnaBios Corporation at the time of completion of work.

## Author contributions

IG, HKK, LK‐N, PEM, AG, NA‐G, TA, JG, DU, HA‐S, and JWA participated in research design. IG, KB, HD, XC, TA, A‐TT, and TI conducted experiments. IG, HKK, DTS, KB, HD, XC, TA, LK‐N, and DU performed data analysis. IG, HKK, DTS, DU, CJR, CC, and JWA wrote or contributed to the writing of the manuscript. All authors critically reviewed and approved the final draft manuscript.

## Supporting information


**Fig. S1.** S1P_2_ internalization by fluorescent microscopy and flow cytometry.
**Table S1.** Listing of donors of cadaver heart tissue used in the GIRK channel activation studies.
**Table S2.** Pharmacologic profiling of etrasimod and other S1PR modulators using cell lines transduced with hS1P_2–5_.

## Data Availability

Upon request, and subject to review, Pfizer will provide the data that support the findings of this study. Subject to certain criteria, conditions, and exceptions, Pfizer may also provide access to the related individual de‐identified participant data. See https://www.pfizer.com/science/clinical‐trials/trial‐data‐and‐results for more information.

## References

[1] Kihara Y , Maceyka M , Spiegel S and Chun J (2014) Lysophospholipid receptor nomenclature review: IUPHAR review 8. Br J Pharmacol 171, 3575–3594. 24602016 10.1111/bph.12678 PMC4128058

[2] Kyoto Encyclopedia of Genes and Genomes (2024) KEGG PATHWAY: ko04071. https://www.genome.jp/entry/ko04071

[3] Camm J , Hla T , Bakshi R and Brinkmann V (2014) Cardiac and vascular effects of fingolimod: mechanistic basis and clinical implications. Am Heart J 168, 632–644. 25440790 10.1016/j.ahj.2014.06.028

[4] Constantinescu V , Haase R , Akgün K and Ziemssen T (2022) S1P receptor modulators and the cardiovascular autonomic nervous system in multiple sclerosis: a narrative review. Ther Adv Neurol Disord 15, 17562864221133163. 36437849 10.1177/17562864221133163 PMC9685213

[5] Blaho VA and Hla T (2014) An update on the biology of sphingosine 1‐phosphate receptors. J Lipid Res 55, 1596–1608. 24459205 10.1194/jlr.R046300 PMC4109755

[6] Walzer T , Chiossone L , Chaix J , Calver A , Carozzo C , Garrigue‐Antar L , Jacques Y , Baratin M , Tomasello E and Vivier E (2007) Natural killer cell trafficking in vivo requires a dedicated sphingosine 1‐phosphate receptor. Nat Immunol 8, 1337–1344. 17965716 10.1038/ni1523

[7] Uhlén M , Fagerberg L , Hallström BM , Lindskog C , Oksvold P , Mardinoglu A , Sivertsson Å , Kampf C , Sjöstedt E , Asplund A *et al*. (2015) Proteomics. Tissue‐based map of the human proteome. Science 347, 1260419. 25613900 10.1126/science.1260419

[8] The Human Protein Atlas (2022) https://www.proteinatlas.org/.

[9] Cartier A and Hla T (2019) Sphingosine 1‐phosphate: lipid signaling in pathology and therapy. Science 366, eaar5551. 31624181 10.1126/science.aar5551 PMC7661103

[10] Pham TH , Okada T , Matloubian M , Lo CG and Cyster JG (2008) S1P1 receptor signaling overrides retention mediated by G alpha i‐coupled receptors to promote T cell egress. Immunity 28, 122–133. 18164221 10.1016/j.immuni.2007.11.017 PMC2691390

[11] Cyster JG and Schwab SR (2012) Sphingosine‐1‐phosphate and lymphocyte egress from lymphoid organs. Annu Rev Immunol 30, 69–94. 22149932 10.1146/annurev-immunol-020711-075011

[12] Tran JQ , Hartung JP , Peach RJ , Boehm MF , Rosen H , Smith H , Brooks JL , Timony GA , Olson AD , Gujrathi S *et al*. (2017) Results from the first‐in‐human study with Ozanimod, a novel, selective Sphingosine‐1‐phosphate receptor modulator. J Clin Pharmacol 57, 988–996. 28398597 10.1002/jcph.887 PMC5516232

[13] Gergely P , Nuesslein‐Hildesheim B , Guerini D , Brinkmann V , Traebert M , Bruns C , Pan S , Gray NS , Hinterding K , Cooke NG *et al*. (2012) The selective sphingosine 1‐phosphate receptor modulator BAF312 redirects lymphocyte distribution and has species‐specific effects on heart rate. Br J Pharmacol 167, 1035–1047. 22646698 10.1111/j.1476-5381.2012.02061.x PMC3485666

[14] Brinkmann V , Billich A , Baumruker T , Heining P , Schmouder R , Francis G , Aradhye S and Burtin P (2010) Fingolimod (FTY720): discovery and development of an oral drug to treat multiple sclerosis. Nat Rev Drug Discov 9, 883–897. 21031003 10.1038/nrd3248

[15] Gräler MH and Goetzl EJ (2004) The immunosuppressant FTY720 down‐regulates sphingosine 1‐phosphate G‐protein‐coupled receptors. FASEB J 18, 551–553. 14715694 10.1096/fj.03-0910fje

[16] Schmouder R , Serra D , Wang Y , Kovarik JM , DiMarco J , Hunt TL and Bastien MC (2006) FTY720: placebo‐controlled study of the effect on cardiac rate and rhythm in healthy subjects. J Clin Pharmacol 46, 895–904. 16855074 10.1177/0091270006289853

[17] Wisler JW , Rockman HA and Lefkowitz RJ (2018) Biased G protein‐coupled receptor signaling: changing the paradigm of drug discovery. Circulation 137, 2315–2317. 29844068 10.1161/CIRCULATIONAHA.117.028194 PMC5984047

[18] Juif PE , Ufer M and Dingemanse J (2019) Cardiodynamic interactions between two S1P(1) receptor modulators in an experimental clinical setting: different pharmacokinetic properties as an opportunity to mitigate first‐dose heart rate effects. Int J Mol Sci 20, 3232. 31266149 10.3390/ijms20133232 PMC6651405

[19] Whalen EJ , Rajagopal S and Lefkowitz RJ (2011) Therapeutic potential of β‐arrestin‐ and G protein‐biased agonists. Trends Mol Med 17, 126–139. 21183406 10.1016/j.molmed.2010.11.004 PMC3628754

[20] Zhu Y , Watson J , Chen M , Shen DR , Yarde M , Agler M , Burford N , Alt A , Jayachandra S , Cvijic ME *et al*. (2014) Integrating high‐content analysis into a multiplexed screening approach to identify and characterize GPCR agonists. J Biomol Screen 19, 1079–1089. 24789006 10.1177/1087057114533146

[21] Billich A , Bornancin F , Dévay P , Mechtcheriakova D , Urtz N and Baumruker T (2003) Phosphorylation of the immunomodulatory drug FTY720 by sphingosine kinases. J Biol Chem 278, 47408–47415. 13129923 10.1074/jbc.M307687200

[22] Kappos L , Radue EW , O'Connor P , Polman C , Hohlfeld R , Calabresi P , Selmaj K , Agoropoulou C , Leyk M , Zhang‐Auberson L *et al*. (2010) A placebo‐controlled trial of oral fingolimod in relapsing multiple sclerosis. N Engl J Med 362, 387–401. 20089952 10.1056/NEJMoa0909494

[23] Calabresi PA , Radue EW , Goodin D , Jeffery D , Rammohan KW , Reder AT , Vollmer T , Agius MA , Kappos L , Stites T *et al*. (2014) Safety and efficacy of fingolimod in patients with relapsing‐remitting multiple sclerosis (FREEDOMS II): a double‐blind, randomised, placebo‐controlled, phase 3 trial. Lancet Neurol 13, 545–556. 24685276 10.1016/S1474-4422(14)70049-3

[24] Peyrin‐Biroulet L , Christopher R , Behan D and Lassen C (2017) Modulation of sphingosine‐1‐phosphate in inflammatory bowel disease. Autoimmun Rev 16, 495–503. 28279838 10.1016/j.autrev.2017.03.007

[25] Kappos L , Bar‐Or A , Cree BAC , Fox RJ , Giovannoni G , Gold R , Vermersch P , Arnold DL , Arnould S , Scherz T *et al*. (2018) Siponimod versus placebo in secondary progressive multiple sclerosis (EXPAND): a double‐blind, randomised, phase 3 study. Lancet 391, 1263–1273. 29576505 10.1016/S0140-6736(18)30475-6

[26] Silverberg JI , Bissonnette R , Kircik L , Murrell DF , Selfridge A , Liu K , Ahluwalia G and Guttman‐Yassky E (2023) Efficacy and safety of etrasimod, a sphingosine 1‐phosphate receptor modulator, in adults with moderate‐to‐severe atopic dermatitis (ADVISE). J Eur Acad Dermatol Venereol 37, 1366–1374. 36695074 10.1111/jdv.18914

[27] Dellon ES , Collins MH , Bredenoord AJ , Philpott H , Biedermann L , Dulcine M , Nguyen‐Cleary T , Su C , Yu J , Cataldi F *et al*. (2023) S455 efficacy and safety of the selective sphingosine 1‐phosphate receptor modulator, etrasimod, in adult patients with eosinophilic esophagitis: primary results from the phase 2 VOYAGE study. Am J Gastroenterol 118, S330–S331.

[28] Dellon ES , Collins MH , Bredenoord A , Philpott H , Biedermann L , Dulcine M , Nguyen‐Cleary T , Su C , Yu J , Tan H *et al*. (2024) 635 efficacy and safety of the selective sphingosine 1‐phosphate receptor modulator, etrasimod, in adult patients with eosinophilic esophagitis over 52 weeks in the phase 2 VOYAGE study. Gastroenterology 166, S‐146–S‐147.

[29] Sandborn WJ , Vermeire S , Peyrin‐Biroulet L , Dubinsky MC , Panes J , Yarur A , Ritter T , Baert F , Schreiber S , Sloan S *et al*. (2023) Etrasimod as induction and maintenance therapy for ulcerative colitis (ELEVATE): two randomised, double‐blind, placebo‐controlled, phase 3 studies. Lancet 401, 1159–1171. 36871574 10.1016/S0140-6736(23)00061-2

[30] Kiyomi K , Caroline L , Lisette A , Kye G and John G (2020) P045 effect of Etrasimod on circulating lymphocyte subsets: data from a randomized phase 1 study in healthy Japanese and Caucasian men. Am J Gastroenterol 115, S12. 10.14309/01.ajg.0000722976.59079.27 33566519

[31] Surapaneni S , Yerramilli U , Bai A , Dalvie D , Brooks J , Wang X , Selkirk JV , Yan YG , Zhang P , Hargreaves R *et al*. (2021) Absorption, metabolism, and excretion, in vitro pharmacology, and clinical pharmacokinetics of Ozanimod, a novel sphingosine 1‐phosphate receptor modulator. Drug Metab Dispos 49, 405–419. 33674268 10.1124/dmd.120.000220

[32] David OJ , Kovarik JM and Schmouder RL (2012) Clinical pharmacokinetics of fingolimod. Clin Pharmacokinet 51, 15–28. 22149256 10.2165/11596550-000000000-00000

[33] Shakeri‐Nejad K , Gardin A , Gray C , Neelakantham S , Dumitras S and Legangneux E (2020) Safety, tolerability, pharmacodynamics and pharmacokinetics of intravenous Siponimod: a randomized, open‐label study in healthy subjects. Clin Ther 42, 175–195. 31926605 10.1016/j.clinthera.2019.11.014

[34] Al‐Shamma H , Lehmann‐Bruinsma K , Carroll C , Solomon M , Komori HK , Peyrin‐Biroulet L and Adams J (2019) The selective sphingosine 1‐phosphate receptor modulator etrasimod regulates lymphocyte trafficking and alleviates experimental colitis. J Pharmacol Exp Ther 369, 311–317. 30872391 10.1124/jpet.118.254268

[35] Winpenny D , Clark M and Cawkill D (2016) Biased ligand quantification in drug discovery: from theory to high throughput screening to identify new biased μ opioid receptor agonists. Br J Pharmacol 173, 1393–1403. 26791140 10.1111/bph.13441 PMC4940816

[36] Kenakin T (2017) A scale of Agonism and allosteric modulation for assessment of selectivity, bias, and receptor mutation. Mol Pharmacol 92, 414–424. 28679508 10.1124/mol.117.108787

[37] Abi‐Gerges N , Indersmitten T , Truong K , Nguyen W , Ratchada P , Nguyen N , Page G , Miller PE and Ghetti A (2020) Multiparametric mechanistic profiling of inotropic drugs in adult human primary Cardiomyocytes. Sci Rep 10, 7692. 32376974 10.1038/s41598-020-64657-2 PMC7203129

[38] Page G , Ratchada P , Miron Y , Steiner G , Ghetti A , Miller PE , Reynolds JA , Wang K , Greiter‐Wilke A , Polonchuk L *et al*. (2016) Human ex‐vivo action potential model for pro‐arrhythmia risk assessment. J Pharmacol Toxicol Methods 81, 183–195. 27235787 10.1016/j.vascn.2016.05.016 PMC5042841

[39] Nguyen N , Nguyen W , Nguyenton B , Ratchada P , Page G , Miller PE , Ghetti A and Abi‐Gerges N (2017) Adult human primary Cardiomyocyte‐based model for the simultaneous prediction of drug‐induced inotropic and pro‐arrhythmia risk. Front Physiol 8, 1073. 29311989 10.3389/fphys.2017.01073 PMC5742250

[40] Wu Q , Mills EA , Wang Q , Dowling CA , Fisher C , Kirch B , Lundy SK , Fox DA , Mao‐Draayer Y and AMS04 Study Group (2020) Siponimod enriches regulatory T and B lymphocytes in secondary progressive multiple sclerosis. JCI Insight 5, e134251. 31935197 10.1172/jci.insight.134251 PMC7098784

[41] Poirier B , Briand V , Kadereit D , Schäfer M , Wohlfart P , Philippo MC , Caillaud D , Gouraud L , Grailhe P , Bidouard JP *et al*. (2020) A G protein‐biased S1P1 agonist, SAR247799, protects endothelial cells without affecting lymphocyte numbers. Sci Signal 13, eaax8050. 32487716 10.1126/scisignal.aax8050

[42] Sandborn WJ , Feagan BG , D'Haens G , Wolf DC , Jovanovic I , Hanauer SB , Ghosh S , Petersen A , Hua SY , Lee JH *et al*. (2021) Ozanimod as induction and maintenance therapy for ulcerative colitis. N Engl J Med 385, 1280–1291. 34587385 10.1056/NEJMoa2033617

[43] Means CK and Brown JH (2009) Sphingosine‐1‐phosphate receptor signalling in the heart. Cardiovasc Res 82, 193–200. 19282351 10.1093/cvr/cvp086 PMC2721649

[44] Zhang J , Honbo N , Goetzl EJ , Chatterjee K , Karliner JS and Gray MO (2007) Signals from type 1 sphingosine 1‐phosphate receptors enhance adult mouse cardiac myocyte survival during hypoxia. Am J Physiol Heart Circ Physiol 293, H3150–H3158. 17766476 10.1152/ajpheart.00587.2006

[45] Means CK , Miyamoto S , Chun J and Brown JH (2008) S1P_1_ receptor localization confers selectivity for G_i_‐mediated cAMP and contractile responses. J Biol Chem 283, 11954–11963. 18296752 10.1074/jbc.M707422200 PMC2335351

[46] Landeen LK , Dederko DA , Kondo CS , Hu BS , Aroonsakool N , Haga JH and Giles WR (2008) Mechanisms of the negative inotropic effects of sphingosine‐1‐phosphate on adult mouse ventricular myocytes. Am J Physiol Heart Circ Physiol 294, H736–H749. 18024550 10.1152/ajpheart.00316.2007

[47] Theilmeier G , Schmidt C , Herrmann J , Keul P , Schafers M , Herrgott I , Mersmann J , Larmann J , Hermann S , Stypmann J *et al*. (2006) High‐density lipoproteins and their constituent, sphingosine‐1‐phosphate, directly protect the heart against ischemia/reperfusion injury in vivo via the S1P3 lysophospholipid receptor. Circulation 114, 1403–1409. 16982942 10.1161/CIRCULATIONAHA.105.607135

[48] Robert P , Tsui P , Laville MP , Livi GP , Sarau HM , Bril A and Berrebi‐Bertrand I (2001) EDG1 receptor stimulation leads to cardiac hypertrophy in rat neonatal myocytes. J Mol Cell Cardiol 33, 1589–1606. 11549339 10.1006/jmcc.2001.1433

[49] Ghosh TK , Bian J and Gill DL (1990) Intracellular calcium release mediated by sphingosine derivatives generated in cells. Science 248, 1653–1656. 2163543 10.1126/science.2163543

[50] Brakch N , Dormond O , Bekri S , Golshayan D , Correvon M , Mazzolai L , Steinmann B and Barbey F (2010) Evidence for a role of sphingosine‐1 phosphate in cardiovascular remodelling in Fabry disease. Eur Heart J 31, 67–76. 19773225 10.1093/eurheartj/ehp387

[51] Blankenbach KV , Schwalm S , Pfeilschifter J and Meyer Zu Heringdorf D (2016) Sphingosine‐1‐phosphate Receptor‐2 antagonists: therapeutic potential and potential risks. Front Pharmacol 7, 167. 27445808 10.3389/fphar.2016.00167 PMC4914510

[52] Brinkmann V , Davis MD , Heise CE , Albert R , Cottens S , Hof R , Bruns C , Prieschl E , Baumruker T , Hiestand P *et al*. (2002) The immune modulator FTY720 targets sphingosine 1‐phosphate receptors. J Biol Chem 277, 21453–21457. 11967257 10.1074/jbc.C200176200

[53] Cohen JA , Barkhof F , Comi G , Hartung HP , Khatri BO , Montalban X , Pelletier J , Capra R , Gallo P , Izquierdo G *et al*. (2010) Oral fingolimod or intramuscular interferon for relapsing multiple sclerosis. N Engl J Med 362, 402–415. 20089954 10.1056/NEJMoa0907839

[54] Selmaj KW , Cohen JA , Comi G , Bar‐Or A , Arnold DL , Steinman L , Hartung HP , Montalban X , Havrdova EK , Cree BAC *et al*. (2021) Ozanimod in relapsing multiple sclerosis: pooled safety results from the clinical development program. Mult Scler Relat Disord 51, 102844. 33892317 10.1016/j.msard.2021.102844

[55] Gon Y , Wood MR , Kiosses WB , Jo E , Sanna MG , Chun J and Rosen H (2005) S1P3 receptor‐induced reorganization of epithelial tight junctions compromises lung barrier integrity and is potentiated by TNF. Proc Natl Acad Sci U S A 102, 9270–9275. 15968000 10.1073/pnas.0501997102 PMC1166603

[56] Vermeire S , Chiorean M , Panés J , Peyrin‐Biroulet L , Zhang J , Sands BE , Lazin K , Klassen P , Naik SU , Cabell CH *et al*. (2021) Long‐term safety and efficacy of Etrasimod for ulcerative colitis: results from the open‐label extension of the OASIS study. J Crohns Colitis 15, 950–959. 33475734 10.1093/ecco-jcc/jjab016 PMC8218705

[57] Jenne CN , Enders A , Rivera R , Watson SR , Bankovich AJ , Pereira JP , Xu Y , Roots CM , Beilke JN , Banerjee A *et al*. (2009) T‐bet‐dependent S1P5 expression in NK cells promotes egress from lymph nodes and bone marrow. J Exp Med 206, 2469–2481. 19808259 10.1084/jem.20090525 PMC2768857

[58] Mayol K , Biajoux V , Marvel J , Balabanian K and Walzer T (2011) Sequential desensitization of CXCR4 and S1P5 controls natural killer cell trafficking. Blood 118, 4863–4871. 21911833 10.1182/blood-2011-06-362574

