# Supplementary material for: Unique pharmacological properties of etrasimod among S1P receptor modulators
Source: FEBS Open Bio. 2024 Nov 20;15(1):108–21. doi: 10.1002/2211-5463.13907 (PMC11705451; doi:10.1002/2211-5463.13907)
Supplement: Supplementary file 1 — Fig. S1. S1P2 internalization by fluorescent microscopy and flow cytometry. Table S1. Listing of donors of cadaver heart tissue used in the GIRK channel activation studies. Table S2. Pharmacologic profiling of etrasimod and other S1PR modulators using cell lines transduced with hS1P2–5. [file FEB4-15-108-s001.docx]

# Supporting Information

Table S1. Listing of donors of cadaver heart tissue used in the GIRK channel activation studies

| Donor number | Donor identifier | Age | Sex | Ethnicity | Body mass index, kg/m^2^ |
| --- | --- | --- | --- | --- | --- |
| 1 | 181202HHA | 50 | F | Caucasian | 19.0 |
| 2 | 181208HHA | 58 | F | Caucasian | 30.1 |
| 3 | 181220HHA | 46 | F | Caucasian | 30.5 |
| 4 | 190114HHA | 26 | F | Caucasian | 19.4 |
| 5 | 190123HHA | 52 | M | Hispanic | 32.6 |
| 6 | 180723HHA | 36 | M | Caucasian | 21.0 |

GIRK, G protein-gated inwardly rectifying potassium.

Table S2. Pharmacologic profiling of etrasimod and other S1PR modulators using cell lines transduced with hS1P_2-5_

| Compound | Assay | S1P_2_ | S1P_3_ | S1P_4_ | S1P_5_ |
| --- | --- | --- | --- | --- | --- |
| Etrasimod | **β-Arrestin** | | | | |
|  | EC_50_, mean (95% CI) [n], nM | NR [4] | NR [4] | 141  (97.5-204) [3] | 63.4  (40-101) [3] |
|  | E_max_, % of S1P | NR | NR | 53.9 | 85.7 |
|  | **GTPγS** | | | | |
|  | EC_50_, mean (95% CI) [n], nM | NR [4] | 6050  (2540-14400) [4] | NR [4] | 63.5  (17-237.7) [4] |
|  | E_max_, % of S1P | NR | 41.5 | NR | 72 |
| Ozanimod | **β-Arrestin** | | | | |
|  | EC_50_, mean (95% CI) [n], nM | NR [4] | NR [3] | 766  (604-971) [4] | 28.8  (12.6-65.6) [4] |
|  | E_max_, % of S1P | NR | NR | 104.6 | 118.7 |
|  | **GTPγS** | | | | |
|  | EC_50_, mean (95% CI) [n], nM | NR [4] | 6820  (3430-13500) [4] | NR [4] | 12.3  (6.8-22) [4] |
|  | E_max_, % of S1P | NR | 52.3 | NR | 109.6 |
| Ozanimod(K) | **β-Arrestin** | | | | |
|  | EC_50_, mean (95% CI) [n], nM | NR [4] | NR [4] | 588  (404-855) [4] | 58.5  (28.7-119) [4] |
|  | E_max_, % of S1P | NR | NR | 89.1 | 115.2 |
|  | **GTPγS** | | | | |
|  | EC_50_, mean (95% CI) [n], nM | NR [4] | NR [4] | NR [4] | 95.8  (38.4-239) [4] |
|  | E_max_, % of S1P | NR | NR | NR | 105.2 |
| Fingolimod(P) | **β-Arrestin** | | | | |
|  | EC_50_, mean (95% CI) [n], nM | NR [4] | 22.3  (10.2-48.5) [4] | 6.9  (3.6-13.5) [4] | 5.8  (4.4-7.7) [4] |
|  | E_max_, % of S1P | NR | 9.1 | 77.6 | 48.4 |
|  | **GTPγS** | | | | |
|  | EC_50_, mean (95% CI) [n], nM | 1680  (660-4290) [4] | 13.8  (7.7-25.4) [4] | 6.1  (2.6-14.2) [4] | 1.07  (0.57-2) [4] |
|  | E_max_, % of S1P | 35.2 | 39.1 | 121 | 69.5 |
| Siponimod | **β-Arrestin** | | | | |
|  | EC_50_, mean (95% CI) [n], nM | NR [4] | NR [4] | 563  (170-1870) [4] | 11  (2.7-44.6) [4] |
|  | E_max_, % of S1P | NR | NR | 92.6 | 106.8 |
|  | **GTPγS** | | | | |
|  | EC_50_, mean (95% CI) [n], nM | NR [4] | NR [3] | 510  (293-887) [4] | 0.54  (0.2-1.5) [4] |
|  | E_max_, % of S1P | NR | NR | 134 | 118.1 |
| S1P | **β-Arrestin** | | | | |
|  | EC_50_, mean (95% CI) [n], nM | 19  (8.1-44.5) [4] | 16.5  (9.4-28.9) [4] | 5.44  NR [4] | 1.4  (0.9-2.4) [3] |
|  | E_max_, % of S1P | 99.6 | 105.8 | 100.7 | 103.8 |
|  | **GTPγS** | | | | |
|  | EC_50_, mean (95% CI) [n], nM | 115  (32.6-405) [4] | 323  (116-900) [4] | 140  (74.1-263) [4] | 49.7  (17.7-139.4) [4] |
|  | E_max_, % of S1P | 107.9 | 113.3 | 100 | 99.9 |

CI, 95% confidence interval; EC_50_, concentration required for 50% of the maximum effect; n, number of tests; NR, no response; S1P, sphingosine 1-phosphate; S1PR, sphingosine 1-phosphate receptor.

**Figure S1.** S1P_2_ internalization by fluorescent microscopy and flow cytometry. **(A)** PathHunter HEK293 cells stably expressing S1P_2_ were stimulated with 5 μM of test compounds for 90 minutes and the presence of S1P_2_ was detected with fluorescent microscopy. **(B)** Concentration–response curves for S1P_2_ internalization as determined by flow cytometry in CHO-K1 cells stably expressing S1P_2_.


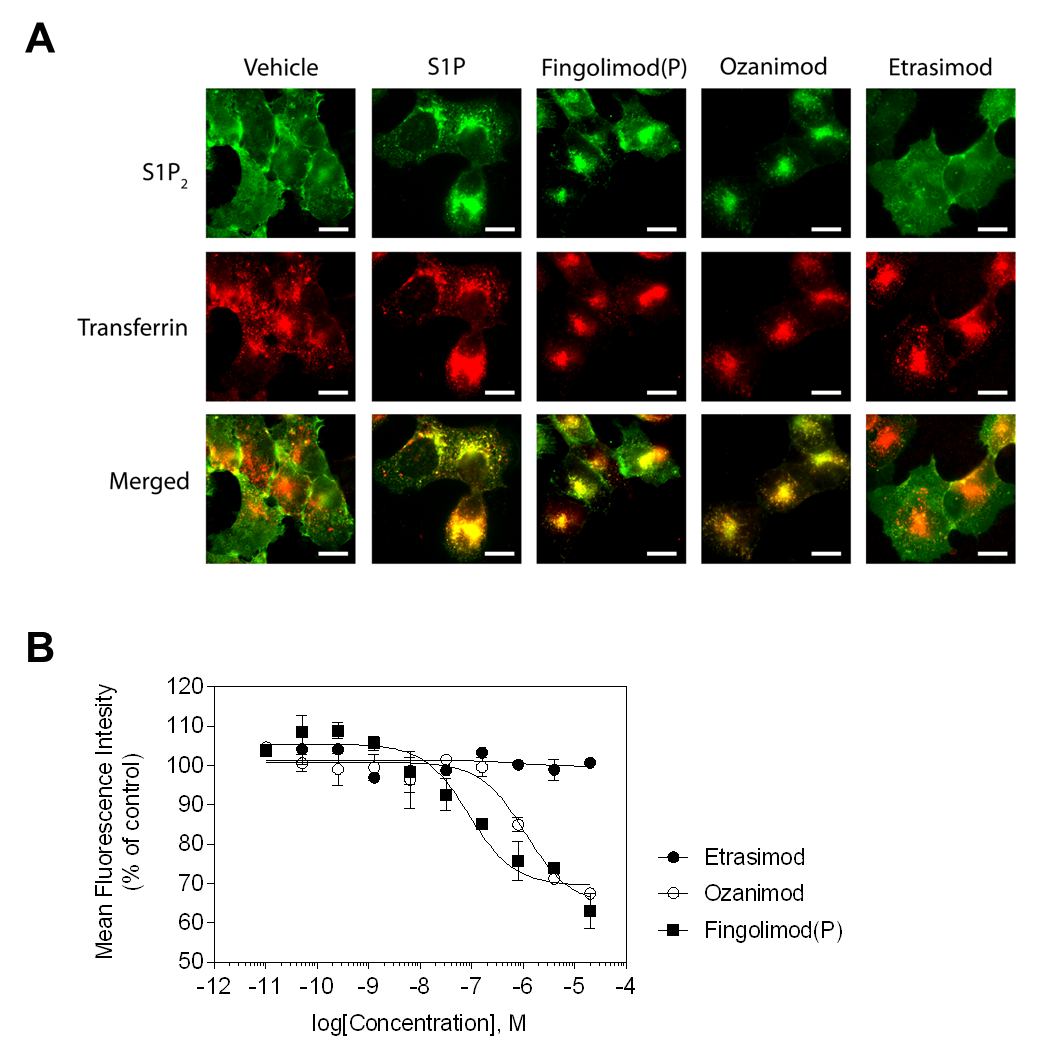


Scale bar, 10 µm. Error bars represent standard deviation (n=3).
